# Superhuman spatial hearing technology for ultrasonic frequencies

**DOI:** 10.1038/s41598-021-90829-9

**Published:** 2021-06-02

**Authors:** Ville Pulkki, Leo McCormack, Raimundo Gonzalez

**Affiliations:** Acoustics Lab, Department of Signal Processing and Acoustics, Aalto University, Espoo, Finland

**Keywords:** Electrical and electronic engineering, Acoustics

## Abstract

Ultrasonic sources are inaudible to humans, and while digital signal processing techniques are available to bring ultrasonic signals into the audible range, there are currently no systems which also simultaneously permit the listener to localise the sources through spatial hearing. Therefore, we describe a method whereby an in-situ listener with normal binaural hearing can localise ultrasonic sources in real-time; opening-up new applications, such as the monitoring of certain forms of wild life in their habitats and man-made systems. In this work, an array of ultrasonic microphones is mounted to headphones, and the spatial parameters of the ultrasonic sound-field are extracted. A pitch-shifted signal is then rendered to the headphones with spatial properties dictated by the estimated parameters. The processing provides the listener with the spatial cues that would normally occur if the acoustic wave produced by the source were to arrive at the listener having already been pitch-shifted. The results show that the localisation accuracy delivered by the proof-of-concept device implemented here is almost as good as with audible sources, as tested both in the laboratory and under conditions in the field.

## Introduction

Human spatial hearing capability^1^ is an important sense for determining the location of events, actions, voices and other sound sources within the surroundings of a listener. The highest level of accuracy of localisation is obtained when one sound source is dominant and has a broad frequency spectrum with fast changes in temporal structure. In a best-case scenario, a localisation accuracy of approximately $1$–$3^\circ$ can be obtained on the horizontal plane in front of the listener^2^. The neurophysiological process for localisation occurs in parallel with analysis of the frequency content of sound, and the perception of an auditory object provides information regarding *what* happened and *where* it happened^3^. The ability to localise sound sources is a fast and potent sensing mechanism to omnidirectionally perceive the surrounding three-dimensional world within a broad frequency range.

Although the sense of hearing is sensitive to a wide range of frequencies, there is an upper frequency limit. This limit is approximately 20 kHz for young human subjects and is gradually lowered with increasing age. The frequencies above 20 kHz are commonly referred to as ultrasonic frequencies. Many animals, such as bats, rodents, insects, reptiles and amphibians produce strong vocalisations in the ultrasonic range^4^, and man-made devices may also generate ultrasonic sounds in their normal or abnormal operation; such as gas leaks in pipes^5^. Ultrasonic signals can be brought to audible frequencies using signal processing techniques, for example, bats are often monitored using specific detectors^6^, which can play back the down-shifted sound through a miniature loudspeaker. However, while the sounds they produce are audible to the listener, such devices do not permit the perception of the direction of ultrasonic sound sources.

The aim of this study was, therefore, to develop a technology to render ultrasonic frequencies audible within the range of human hearing, while simultaneously allowing the directions of the ultrasonic sources to be perceived by the listener in a real acoustic environment. For this task, we propose the use of a miniature head-mounted ultrasonic microphone array, accompanied by parametric spatial audio reproduction of the down-shifted sounds over headphones. The authors are not aware of any previous scientific work on this topic. Specifically, the techniques developed in this article show how to obtain headphone signals from an ultrasonic array of microphones with low latency, in such a manner that the directional information of ultrasonic sources is imprinted onto a signal following a pitch-shifting operation.

In general, human spatial hearing acts as a signal analyser; estimating the most probable locations of perceived sources. This is based on directional cues arising from the acoustical interaction between the listener and the arriving wave front. More specifically, the directional cues have been found to be the differences between the ear canal signals (interaural time and level differences), ear canal spectra (monaural cues) and the effect of head motion on directional cues (dynamic cues)^2,7^.

A trivial solution for auralizing and localizing ultrasonic sounds could involve placing an ultrasonic microphone next to each ear, shifting the ultrasonic frequency content down to the audible range, and then delivering those down-shifted microphone signals to the respective ears of the listener. A less drastic version of such processing is utilised by certain hearing aids^8^, where high frequencies that are audible to normal-hearing subjects, but inaudible to those who are hearing-impaired, are shifted down to the audible range of the wearer. However, such an approach is problematic in terms of the preservation of directional cues, since the acoustic filtering effects due to the diffraction^9^ caused by the head and pinna vary substantially with frequency. Therefore, the directional cues delivered by the pitch-shifted signals do not carry the appropriate information related to the source direction that a human subject would have evolved and grown accustomed to.

Another solution could be to build two ultrasonic microphones into the ear canals of a miniature model of a human head^10^. For example, if a ratio of 1:8 change of pitch is targeted, one could manufacture a 1:8 miniature replica. This replica could then be mounted on top of the head of the listener, and their ultrasonic signals pitch-shifted to audible frequencies and relayed to headphones worn by the listener. However, although through this system the interaural level differences of sound could potentially be reproduced appropriately, the interaural time difference cues would not; since the small distance between the ears of the miniature model would not produce the arrival time differences that would occur naturally between the ears of a real human head. This approach is also limited to a fixed ratio of pitch-shifting, and if some other degree of shifting was then required, a new miniature replica of the listener would need to be manufactured.

A third technique that, in principle, could be used for the task, would be to employ a small array of ultrasonic sensors and to utilise Ambisonics^11^ methods. However, such sensor arrays would require specialised manufacturing, and the authors are not aware of any such device constructed in practice. For example, a tetrahedral array, which is commonly employed for first-order Ambisonics techniques, would require four flat-response ultrasonic capsules positioned in an array with an inter-microphone distance of less than 2 mm. This would be required in order to push the upper frequency limit of the system, also referred to as the spatial aliasing frequency, to 50 kHz. However, assuming that such a device could be constructed, Ambisonics rendering could be employed to first obtain a set of signals for intermediate spherical arrangement of reproduction directions^11^, with pitch-shifting operations then applied to each signal. This would then be followed by the reproduction of each down-shifted signal over headphones using the appropriate respective binaural filters.

A further technical difficulty with the three approaches postulated thus far, relates to the problem that pitch-shifting operations significantly modify the original signals, and thus a number of signal-dependent quality defects can occur. These signal artefacts could also be further compounded by the need to apply the pitch-shifting processing onto more than one signal. Additionally, since real-time operation and low latency is required to maintain the dynamic cues, the pitch-shifting operations should not be computationally prohibitive or employ long temporal windows. Consequently, there is no guarantee that the directional cues relating to a miniature dummy head would be preserved during pitch-shifting, or if pitch-shifted intermediate Ambisonic decoded signals would still produce a clear spatial image.

To the best of the authors’ knowledge, there has been no prior scientific work which attempts to make ultrasonic sound sources both audible and localisable for a listener. However, ultrasonic sound-fields have been analysed for the purpose of visualisation. For example, a miniature array has been used to analyse the direction-of-arrival of the sounds created by bats^12^. Gas leaks in pipes also produce strong ultrasound emissions, and ultrasonic microphone arrays have been employed to find such leaks^5^. In these approaches, the aim is to visually, rather than audibly, guide the user towards the locations of ultrasonic sound sources, by presenting the analysed directional information to the user via a visual display.

## Ultrasonic super-hearing device

**Figure 1:**
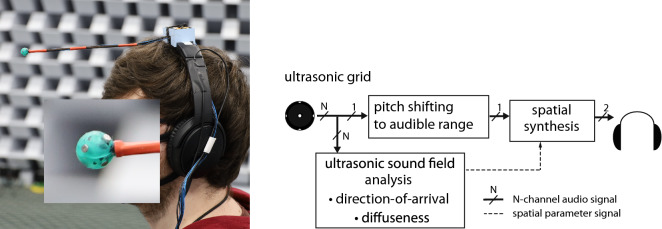
Left: Ultrasonic super-hearing device. Right: Signal processing chain for the device.

The proposed device is based on the application of time-frequency-domain parametric spatial audio techniques, which have been previously developed for the enhancement of sound-field reproduction and compression of spatial sound scenes^13^. The methods first employ spatial analysis techniques over time and frequency, in order to extract spatial parameters of sound-field from an array of microphones. These analysed parameters are then subsequently used to synthesise the audio signals for the target reproduction setup. The processing stages of the proposed technique are depicted in Fig. 1. Note that each signal from the microphone array is first transformed into the short-time frequency domain, and the spatial analysis is conducted for each time-frequency tile independently. The parameter representing the most prominent direction-of-arrival is defined as a unit vector pointing to the direction of arriving sound $\hat{\mathbf {r}}_{\rm {DoA}}(t,f)$, where *t* and *f* are time and frequency parameters, respectively. The second analyzed parameter corresponds to the diffuseness of the sound-field, defined as a real-valued number $\hat{\psi }(t,f) \in [0,1]$, where ideally $\hat{\psi }=0$ is obtained for sound-fields comprising a single plane wave, and $\hat{\psi }=1$ is obtained for a purely diffuse-field or for fields where several sources are active at the same time.

One of the sensor signals is then also modified via a pitch-shifting or down-modulating method, in order to bring captured ultrasonic sounds down to the audible frequency range. This modified signal is first attenuated depending on analysed diffuseness, and subsequently spatialised for playback over headphones, by using the appropriate binaural filters corresponding to the analyed direction-of-arrival parameters. In practice, the system reproduces sound when a single source is dominant, and de-emphasises all other portions of signal. Non-individual head-related transfer function (HRTF) digital filters^14^ were employed for the spatialisation for the device implemented in here.

In the current implementation, the direction-of-arrival was analysed with the rate of at least 40  Hz, with higher rate at higher frequencies. The direction estimates were also averaged over frequency, because it was found that the averaging produced a more stable spatialisation. This is a limitation of the current system, which, nevertheless, did not impede its use. Note that the specific ultrasonic environments explored in this study seldomly comprised more than a single sound source at any one time. However, it should be highlighted that the system also supports frequency-dependent operation, which could improve performance for multi-source scenarios; provided that the sound sources do not overlap significantly in both time and frequency.

In principle, any pitch-shifting technique may be employed, provided that the frequency-dependent parameters analysed from the ultrasonic sound-field are mapped correctly to the frequency scale of the pitch-shifted signal. Since the spatial parameters are averaged over frequency in the currently employed configuration of the device, the frequency mapping is not required in this case. Instead, each time frame of the pitch shifted signal is spatialised according to a frequency-averaged direction. The pitch-shifting technique used for the application targeted in this article should be capable of large pitch-shifting ratios, while also operating within an acceptable latency. Based on these requirements, the phase-vocoder approach^15,16^ was selected for the real-time rendering in this study, due to its low processing latency and acceptable signal quality with large pitch-shifting ratios. However, the application of other pitch-shifting methods is also demonstrated with recordings processed off-line and described in the Results section.

In summary, the proposed processing approach permits frequency-modified signals to be synthesised with plausible binaural and monaural cues, which may subsequently be delivered to the listener to enable the localisation of ultrasonic sound sources. Furthermore, since the super-hearing device turns with the head of the listener, and the processing latency of the device was constrained to 44 ms, the dynamic cues should also be preserved. Note that the effect of processing latency has been previously studied in the context of head-tracked binaural reproduction systems, where it has been found that a system latency above 50–100 ms can impair the spatial perception^17,18^. Therefore, it should be noted that a trade-off must be made between: attaining high spatial image and timbral quality (which are improved through longer temporal windows and a higher level of overlapping) and having low processing latency (which relies on shorter windows and reduced overlapping). The current processing latency has been engineered so that both the spatial image and audio quality after pitch-shifting, as determined based on informal listening, remain reasonably high.

One additional advantage of the proposed approach is that only a single signal is pitch shifted, which is inherently more computationally efficient than pitch-shifting multiple signals; as would be required by the three alternative suggestions described in the Introduction section. Furthermore, the imprinting of the spatial information onto the signal only after pitch-shifting, ensures that the directional cues reproduced for the listener are not distorted by the pitch-shifting operation. The requirements for the size of microphone array are also less stringent compared to the requirements for an Ambisonics-based system. In this work, an array with a diameter of 11 mm was employed, which has a spatial aliasing frequency of approximately 17 kHz. This therefore prohibits the use of Ambisonics for the ultrasonic frequencies with the present array. By contrast, the employed spatial parameter analysis can be conducted above the spatial aliasing frequency; provided that the geometry of the array is known and that the sensors are arranged uniformly on the sphere.

The proof-of-concept device, which was built to demonstrate and evaluate the concept presented in this paper, is shown in Fig. 1, and further detailed in the Methods section. A spherical array comprising 6 microphones, with frequency responses up to approximately 100 kHz, was built and attached to the collar of the headphones of the listener using a 23 cm long probe. The probe protrudes directly infront of the subject, in order to mitigate acoustical shadowing effects for ultrasonic sources located in close frontal proximity to the subject; for example, hand-held ultrasound emitting objects, such as leaking pipes or rubber tyres. Note that when the subject rotates their head, the nominal position of the head relative to a sound source remains fixed. However, the array attached to the probe moves laterally in accentuated left-right directions along an arc. Therefore, the relative azimuth angle $\theta$ of a source on the horizontal plane from the perspective of the head position is different to the corresponding azimuth angle $\theta _{\rm {p}}$ from the array position,

1 $$\begin{aligned} \theta _{\rm {p}} = \arctan {\frac{ \sin {\theta }}{\cos {\theta } - r/R}}, \end{aligned}$$

where *R* is the distance of the source, and *r* is the length of the probe. This phenomenon diminishes with increasing source distance, and is most prevalent when sources are in very close proximity to the listener. Owing to this relative angle mismatch; nearby sources, which originate either to the left or right of the listener, appear somewhat further to the left or right from the perspective of the array compared to that of the head. For example, for a source at a distance of 1.5 m, this biasing effect for a source in $\pm 30^\circ$ of azimuth is approximately $5^\circ$ towards the lateral direction. Therefore, this effect should not have a significant influence on source localisation when using the device, as the human accuracy of localisation is of the same order^2^.

However, the lateral biasing effect is still hypothesised to aid localisation of sound sources located close to the listener. Note that humans commonly localise sound sources based on rotating their heads left and right until a source is perceived as being directly in line with the median plane, which is where the directional accuracy of the human auditory system is highest. The directional biasing caused by the probe therefore creates a so-called “magnifying glass” effect, whereby source direction perception is slightly biased away from the true source positions when they are located to the left or right side of the subject. Therefore, azimuthal head rotations may become a more powerful perceptual tool when attempting to ascertain the true source directions using the device in this particular configuration.

The functionality of the system is evaluated in two stages. A formal listening test quantifies the accuracy of source localisation obtained with the system in the frontal horizontal plane. The performance of the system in informal use is demonstrated with videos under both laboratory and field conditions.

## Results

### Formal listening test

The performance of the system was quantified using a formal subjective listening test, where eight ultrasonic sources were mounted on an arc spanning $28^\circ$ from the viewpoint of the subject. In the test, one of the sources produced ultrasonic sound, and the subjects were asked to indicate which source it was. This was repeated 66 times over randomly selected sources. In the analysis of the data a systematic individual bias was observed. However, such a bias term was expected, since the heads of people are usually asymmetric, and subsequently the headphones to which the microphone array was mounted are oriented slightly differently with each subject. The bias was known to the authors, who compensated for it in informal testing simply by bending the probe left and right until the perceived direction matched-up with the actual direction. However, in order to simplify the procedure, the probe was not manually adjusted. Instead, the individual bias was compensated in the analysis of the subject’s results. The biases of 12 subjects varied between $-7.3^\circ$ and $7.2^\circ$ quite evenly.

**Figure 2:**
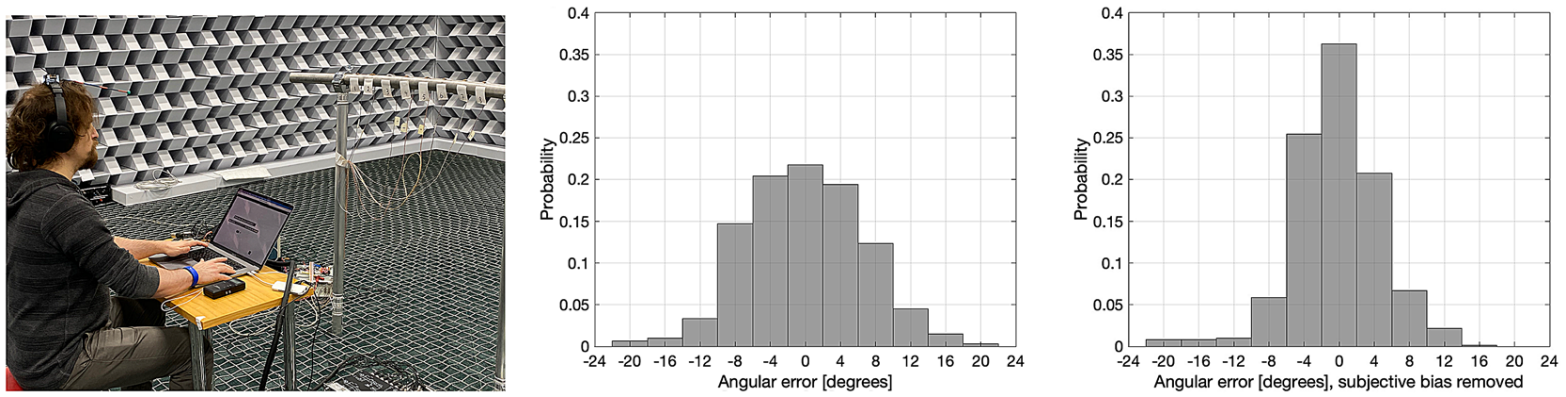
Left: The listening test arrangement. Centre: Distribution of azimuth angular errors in identification of sources, $N=12$, 66 repetitions. Data plotted with individual bias mainly caused by the orientation of the head-worn device. Right: Data plotted after removal of individual bias.

The distribution of angular errors in identifying the sources is shown in Fig. 2, with the individual bias being included and removed. The distribution is demonstratively narrower when the bias effect is removed. The standard deviation of angular errors is $6.3\pm 0.7^\circ$ with raw data, and $4.1\pm 0.5^\circ$ with bias-compensated data, where the value of deviation with a 95% confidence interval is presented. If the subjects had indicated the sources by only guessing with equal probability assigned to each response direction, the standard deviation would have been $11.6^\circ$, which was computed via numerical simulation of the test. It can thus be stated, that since the subject selected the source on a narrow spatial sector around the direction of the source, the device makes it possible to localise ultrasonic sources.

The mean absolute azimuth error of localisation was $5.3^\circ$ and $3.7^\circ$ for the raw data and bias-compensated data, respectively. For reference, the human accuracy of directional perception, of audible sound sources, is of order of around 1–$3^\circ$ in the front of the listener in optimal conditions^2^. This suggests that the localisation accuracy obtained through the currently implemented super-hearing device is somewhat lower than the accuracy of directional hearing. Note that the aforementioned “magnifying-glass'' effect could also be exaggerated based on manipulating the analysed direction-of-arrival values, which could potentially further improve the localisation accuracy of sources directly in front of the listener.

Non-individualised HRTF synthesis was employed in the super-hearing device for the listening experiment, where the directional cues are imposed upon the pitch-shifted signal using a binaural-mannequin HRTF data set. This processing, therefore, produces slightly different cues to what the listener have otherwise been used to when encountering sound sources in natural environments. In^19,20^ it has been found that the mean absolute error of localisation on the horizontal plane with non-individualised HRTF synthesis was approximately $15^\circ$ at first, and around 8–$10^\circ$ with longer adaptation to the used HRTF set. The results of the listening tests in the current study suggest that notably higher accuracy is obtained with the super-hearing device, which can be hypothesised to be caused by the fact that in current study the listeners were tracking the source with head rotations until it was in the front, where the accuracy of human hearing is the best, while referenced works studied the accuracy more widely on the frontal horizontal plane. Furthermore, the reported “magnifying-glass” effect of the device further may also aid in finding the source.

### Monitoring environments with ultrasonic sources

The device has been applied to various in-situ to acoustical scenarios involving ultrasonic sources. A video camera was also mounted on top of the headphones of the listener, and video footage, along with a headphone audio track demonstrating the usage of the super-hearing system under real life conditions, has been produced and is available in the form of supplementary video clips appended to this article.

To demonstrate the hearing-based localisation of leaks in pressurised pipes, two different kinds of leaks are shown in Supplementary Video 1. The first involves a puncture hole, made with a pin, in a relatively rigid PVC tube filled with approximately 3–4 bar air pressure. The resultant leak is audible and localisable in the video. To demonstrate the audibility of smaller leaks, a pin hole was made in a bicycle tyre inner tube. The leak is also audible and localisable to the user of the device; especially when the array is placed a short distance from the tyre; as demonstrated in the video. Note that in both cases, the sound of the leak is not audible without the use of the super-hearing device.

The setup for the listening test reported above is also demonstrated; where sources 2 and 7 are emitting sounds in sequence, and the listener turns their head towards the source. This is demonstrated in Supplementary Video 2.

The authors also listened to natural environments on a number of occasions using the system, and bat sounds are presented in Supplementary Video 3 from two of these sessions. The super-hearing device makes the locations and flying directions of the bats audible, and in the case of close-by flight, it is also possible to perceive the changes in distance.

In principle, the proposed method can be used with any pitch-shifting method, but only the phase-vocoder approach was implemented for real-time processing. However, a number of other pitch-shifting methods were also employed based on off-line rendering. Sound examples of different spectral and temporal structures, which were all recorded with the proposed device, have been rendered to a binaural audio track using four different pitch-shifting methods in Supplementary Video 4. Note that the pitch-shifting in this demonstration has been conducted using off-line implementations, which are therefore not necessarily representative of the sound quality that could be attained with real-time operation. However, even with off-line processing many of the techniques have difficulties in reproducing the frequency sweeps for listening test, or in reproduction of the dog whistle. In turn, the phase vocoder approach used in the current study provides meaningful results for all different signal types. Nevertheless, the renderings do demonstrate that the perceived direction of the sources is similar across the different pitch-shifting techniques; thus, indicating that the proposed method may also be used with different pitch-shifting methods.

## Discussion

Although the system represents only the first prototype built for the task, the results are promising; especially when considering that the microphones were not explicitly designed for ultrasonic frequencies, and parameters of the system were not specifically optimised. The microphones were developed for use in audio devices, such as hearing aids, and their sensitivity to ultrasonic frequencies is largely fortuitous. The microphones did, however, demonstrate increased background noise near 55 kHz, which negatively affected the analysis results in that frequency region. However, broad-band stimuli and other frequency regions other than 50–60 kHz are still correctly localisable using the device, due to the spectral averaging conducted by the system and also physiologically through spatial hearing.

## Conclusions

In this article, a technique is proposed for real-time listening of temporally isolated ultrasonic sources; intended for subjects with normal binaural hearing. The proposed approach not only renders an ultrasonic sound source audible to humans, but also permits the listener to localise it in the correct direction. The technique relies on the use of a miniature ultrasonic microphone array for two purposes: (1) to analyse the spatial properties of an ultrasonic sound-field depending on time and frequency; and (2) to capture a monophonic signal, which is subsequently transformed into the audible frequency range via pitch-shifting methods. The portion of pitch-shifted signal analysed to originate from a single source is then spatially synthesised to headphones worn by the user, where the direction of pitch-shifted sound is perceived based upon the estimated source direction. Since the spatial synthesis of sound is performed after the pitch-shifting operation occurs, the directional cues are not distorted by signal processing artefacts that may arise during the pitch-shifting.

A proof-of-concept device was built using an array of six ultrasonic microphones flush mounted on a rigid sphere. The array was affixed to the collar of a pair of headphones via a probe, which protruded outwards directly in-front of the listener. This probe placement serves to mitigate acoustical shadowing effects arising due to ultrasonic sources located in close proximity and in front of the listener. The probe also creates a small directional bias towards the ipsilateral direction for nearby sources, which aids in finding the head orientation where the source is directly in front. This so-called “magnifying glass” phenomenon represents a feature of the device which may assist localisation. A formal listening test was conducted under laboratory conditions using ultrasonic frequency swept sounds. The results based upon subjects with normal binaural hearing faculties, show that the directional accuracy for this source identification task, for those sources located in front of the listener, is within a few degrees.

The practical usability of the system is also demonstrated via video format and presented with the accompanying first-person viewpoint video footage; which shows the proof-of-concept device being used to capture and render a variety of laboratory and natural ultrasonic sound-fields. These demonstrations show that the system allows the in-situ listener to perceive the directions of ultrasonic sound sources in real-time.

## Methods

### Ultrasonic microphone array

**Figure 3:**
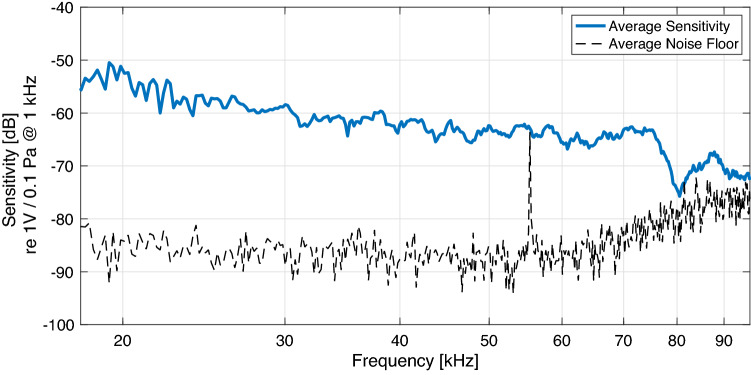
Average sensitivity and noise floor for the 6 sensors that were mounted on the spherical baffle.

The ultrasonic microphone array comprises 6 sensors flush mounted and uniformly distributed on the surface of a rigid sphere of radius 5.5 mm as depicted in Fig. 2. The sensors used were Knowles FG-23629-D65 electret microphones with sound pressure sensitivity higher than the noise floor up to about 100 kHz. A smooth spherical enclosure was produced using a Digital Light Processing (DLP) 3D printer with a layer resolution of 50 $\upmu$m. The sensors in the array were found to have a nominal sensitivity of $-63$ dB ($+/-$ 4 dB) at 40 kHz (re 1 V / 1 Pa @ 1 kHz) and a noise floor of $-85$ dB as depicted in Fig. 3. The array was mounted on Bose QuietComfort II headphones. The analogue processing also involves a first-order high-pass filter at 12 kHz to reduce the level of audible sounds that are not necessary for the intended processing.

### System for on-line binaural reproduction of ultrasonic spatial sound

The complete system consisted of the ultrasonic array routing its six channels of audio to a RME Fireface UCX interface with a sampling rate of 192 kHz. The audio was processed through a custom developed audio plug-in hosted by the audio software REAPER. The processing signal chain comprised two stages: analysis and synthesis.

In the analysis stage, a complex quadrature-mirror filterbank (QMF) is employed to first divide the input signals into 512 uniformly spaced frequency bands, which are then analysed independently^21^. These time-frequency domain signals $\mathbf {x}(t,f) = [x(t,f),...,x_Q(t,f)] \in \mathbb {C} ^{Q \times 1}$ are denoted with *t* and *f* to represent the down-sampled time and frequency indices, respectively. Given that the intended operating range of the system is above the spatial aliasing frequency of the array, the direction-of-arrival (DoA) unit vector, $\hat{\mathbf {r}}_{\rm {DoA}}(t,f)$, is estimated using the sensor-amplitude driven space-domain approach proposed in^22^. This relies on first determining the instantaneous DoA estimates as

2 $$\begin{aligned} \hat{\mathbf {r}}_{\rm {DoA}}(t,f) = \sum _{q=1}^Q |x_q(t,f)| \mathbf {n}\left( \Omega _q\right) , \end{aligned}$$

where $\mathbf {n}(\Omega _q) \in \mathbb {R}^{3 \times 1}$ are Cartesian unit vectors describing the direction of each sensor, *q*. Note that the spherical array causes prominent acoustical shadowing of sound waves and the amplitude $|x_q(t,f)|$ is highest on the side of arrival, and lowest on the opposite shadowed side. When the direction vectors of the sensors are weighted with the amplitude values and summed, the resulting vector points to the most prominent direction-of-arrival of sound. Note that since these estimates do not rely on inter-sensor phase relations, they are unaffected by spatial-aliasing. The DoA analysis approach is described in more detail in^23^, where also the accuracy obtained with it is quantified with a planar array mounted on a rigid cylinder, and compared to standard methods of DoA analysis without rigid baffle. The shadowing-based analysis method provides the same accuracy with other methods in a free-field. However, when there are additional sources or diffuse noise, the method produces higher errors than other methods. The main advantages of the implemented method are that it yields high accuracy for single-source-dominant plane waves, has low computational requirements, and the method can operate within short time windows.

For this study, the estimated DoA vectors were averaged over time using a one-pole filter of coefficient value 0.7, and also over frequencies between 20 kHz and 55 kHz; since the majority of the tested scenarios and the listening test involved only a single dominant sound source at time. However, it is noted that fully frequency-dependent operation is also possible, which could allow the method to resolve the directions of multiple sound sources; provided that they do not overlap substantially in time and frequency. This averaging frequency range was selected as it is above the human upper frequency limit of hearing, and below the noise floor tonal aberration depicted in Fig. 3; it is also where the noise floor begins to trend upwards and the mic sensitivity downwards. This averaged vector of observed DoAs, denoted by $\hat{{\varvec{\rho }}}$, is then employed to determine a diffuseness measure^22^ corresponding to the spherical variance^24^ of the estimates

3 $$\begin{aligned} \hat{\psi } = 1-||\hat{{\varvec{\rho }}}||. \end{aligned}$$

The estimated spatial parameters and the signal corresponding to the sensor located on the top-side of the array are then passed to the synthesis processing stage. Here, the sensor signal is pitch shifted using the phase-vocoder approach^15,16^, by a pitch-shift ratio *r* of 1:8 (3 octaves). Therefore, for example, the signal energy in the frequency region between 20 and 80 kHz now appears between 2.5 and 10 kHz. However, it should be noted that certain aberrations imposed upon the signal during the pitch-shifting operation may, in practice, unintentionally produce energy in other frequencies. Three octaves also represents a particularly drastic pitch-shifting ratio for any pitch-shifting algorithm.

The diffuseness estimate is then employed to modulate the amplitude of the pitch-shifted sensor signal, for the purpose of improving the reproduced signal quality by deactivating it during periods of when the sound-field is not dominated by a single source

4 $$\begin{aligned} p(t,\hat{f}) = (1-\hat{\psi }) \mathcal {P}\left[ x_p(t,f)\right] , \end{aligned}$$

where *p* denotes the pitch-shifted signal, $\hat{f}$ and *f* are pitch-shifted and original frequencies, respectively, $x_p$ is one of the *Q* microphone array sensor signals, and $\mathcal {P}[.]$ denotes a pitch-shifting operation on the enclosed signal. Moreover, $\hat{f} = r f$.

Only the frequencies of pitch shifted signal that were originally ultrasonic are then binauralised. This means that the signal is positioned in the average analysed direction by convolving it with a digital filter corresponding to the head-related transfer function^14^ (HRTF), $\mathbf {h}\in \mathbb {C}^{2 \times 1}$

5 $$\begin{aligned} \mathbf {b}(t,\hat{f}) = \mathbf {h}\left( \hat{\mathbf {r}}^{(avg)},t,\hat{f}\right) p(t,\hat{f}), ~~~~~~~~\hat{f} \in \{(20~000)r, (80~000)r\}, \end{aligned}$$

where $\hat{\mathbf {r}}^{(avg)}$ is the source direction estimate averaged over *f*, and $\mathbf {b}(t,\hat{f})$ are the binaural signals sent to the headphones of the listener after the appropriate inverse time-frequency transform has been conducted. The HRTF set used for the binaural synthesis consisted of 836 responses simulated with the geometry of Kemar binaural mannequin. As commonly applied in practice, triangular interpolation of the loaded HRTF data was employed to obtain interpolated HRTFs in the estimated direction over time.

This is similar to the parametric spatial audio method known as Directional Audio Coding^25^, where, although the sound is not pitch-processed before reproduction; frequency-dependent spatial parameters are also employed during the synthesis of the output signals. The frequency averaging used in the realised proof-of-concept device described here is a detail of this implementation that was found to slightly improve sound quality and help stabilise the spatialisation. The main drawback of the averaging process is that if several source signals with different spectral content and similar amplitude exist at the same time, the resultant analysed direction-of-arrival parameter will not correctly reflect the direction of any of the sources. However, this was not found to be a major problem in this study; as such cases were rare in the ultrasound scenarios tested so far, and it would appear that natural sound environments are typically not crowded in the ultrasonic frequency range. Nevertheless, if the presence of simultaneous sound sources is found to cause quality defects in a given application, the same processing principles presented would still apply, except without frequency averaging of the parameters.

The developed system operates within reasonable computational constraints. The system latency includes the filterbank latency of 24.0 ms (9 hops of overlapping concurrent frames of length 512 samples at 192 kHz), and the latency incurred with the pitch-shifting of 20.0 ms (3840 samples at 192 kHz), an overall total of 44.0 ms. This was found to be an acceptable compromise between signal quality and the responsiveness of the system. Note that more information regarding the software implementation and array construction (including pre-compiled binaries, source code, and CAD files), can be found on the companion web-page^26^.

### Subjective test

The aim of the subjective listening test was to characterise the performance of the super-hearing system by ascertaining the perceived localisation accuracy on the frontal horizontal plane.

#### Setup and stimuli

The subjective test was conducted in a large anechoic chamber. Eight ultrasonic sources were mounted using fabric tape on a curved piece of metallic tube, with a distance of 152–154 cm from the subject. A clearly visible numerical tag was attached just below each source, with numbers 1 to 8 from left to right. The angular separation between adjacent sources was $4^\circ$ from the viewpoint of the subject. The subject was seated on a stool behind a small table where a laptop computer was placed, which controlled the listening test procedure; as shown in Fig. 2.

The ultrasonic sources used were PC4WL co-fired piezoelectric actuators from Thorlabs of dimensions 4.5 $\times$ 3.5 $\times$ 5.0 mm. The signal utilised in the test was a repeated sequence of 50 ms linear frequency chirps from 22  to 44 kHz, synthesised with a GW Instek AFG-2225 function generator. The signal was boosted with an A23 Parasound amplifier and offset to non-negative values through the addition of a DC voltage source. The signal was then routed to each piezo driver using a custom Arduino-controlled relay circuit, which was further controlled by a laptop running Max, a graphical programming software.

**Figure 4:**
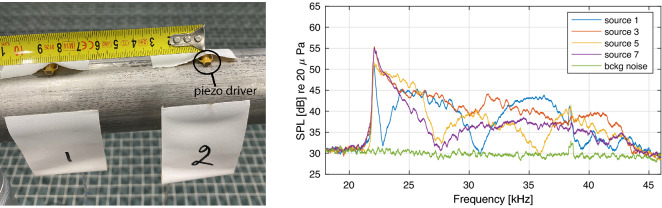
Left: Two piezo drivers of the listening test setup shown in Fig. 2, which were used as ultrasound sources in the test. Right: Frequency spectra of four ultrasonic sources used in the listening test averaged in frequency with 200 Hz wide rectangular window.

The spectral content of the sound present when the drivers were active, was measured with a G.R.A.S 46BF 1/4'' microphone; with four exemplary spectra shown in Fig. 4. The sources produced somewhat different spectra, and all of them reproduced a spectral peak near 22 kHz. The sound was at a 5–30 dB higher level than the noise floor produced by the cooling fans of the devices, present in the anechoic laboratory, used for the tests. The presence of the background noise was not seen as being detrimental; as it would mask audible distortion components that could theoretically be produced by piezo drivers.

#### Procedure

The study complies with the Declaration of Helsinki and was approved by the Research Ethics Committee of Aalto University. Subjects entering the listening test, were provided with written instructions describing the nature of the test and the user interface operation. They also gave written informed consent.

To demonstrate the presence of ultrasonic sounds, one of the sources was turned on and off, and its signal was captured on a Petterson Elektronik D200 ultrasound detector. The subjects were asked if they could discern the sound of the source without the detector, and all of them asserted that they could not hear the sound.

The listeners were then given a short 3–5 min period to become familiar with the super-hearing system. They listened to sources using the device, and were permitted to freely switch between the eight sources. The listeners were thus exposed to the timbres of the sources, and it can be assumed that they adapted at least partly to the non-individual HRTFs used for the binauralisation; since the adaptation to non-individual HRTFs has been found to occur even after a short amount of time in a previous study^19^.

In the subsequent formal listening test, the sound emanated from a single driver selected randomly and the subjects indicated which driver they believe was active by pressing the corresponding number key on the laptop keyboard. The leftmost and rightmost drivers were not used in the test to avoid potential bias effects in the results. Once the subject had indicated the position, the sound was stopped. A new driver was then initiated when the subject pressed return on the keyboard. This procedure was conducted 70 times, whereby the first four trials were for familiarisation purposes. The total duration of the test was between 20–30 min for all listeners.

#### Results

A total of 12 subjects participated in the formal listening test; not including the author who implemented the listening test. Each indication of an active source produced the result

6 $$\begin{aligned} \theta _{ij} = \theta _0 - \theta _1, \end{aligned}$$

where $\theta _0$ and $\theta _1$ are the azimuth directions of the true and indicated sources, respectively, *i* is the index of subject and *j* denotes the repetition.

Since the super-hearing device was mounted on headphones, the heads of the listeners are somewhat asymmetric, and slightly different positioning of the device was possible across participants, an individual bias effect was expected. The biases of the subjects were removed from the data with

7 $$\begin{aligned} \hat{\theta }_{ij} = \theta _{ij} - \beta _i \end{aligned},$$

where $\beta _i$ is the individual bias term computed as an average from $\theta _{ij}$ values of subject *i* corresponding to the two centremost drivers. The absolute error was computed simply as

8 $$\begin{aligned} \phi _{ij}= & {} |\theta _{ij}|, \; {\rm {or}} \end{aligned}$$

9 $$\begin{aligned} \hat{\phi }_{ij}= & {} |\hat{\theta }_{ij}|. \end{aligned}$$

By monitoring the data, it was clear that the subjects most commonly selected the source in their individually-biased direction; followed by the next most probable direction being the source immediately adjacent to this. It was relatively uncommon for a participant to misplace the direction by two or more sources. The individually perceived direction of a source thus had to be at least $4^\circ$ inward from the side of the array, to give the subject the possibility to select a source on both sides of it. For this reason, we omitted the data obtained with sources which were perceived in directions closer than $4^\circ$ from the side of the array. After these omissions, 545 values for ${\hat{\theta }}_{ij}$ were collected in total.

The histograms of the data with and without compensation of individual bias are plotted in Fig. 2, where it can be observed that the distributions obtained resemble normal distributions; although the validity of this assumption could not be verified by statistical tests, due to the relatively small data size. Nevertheless, the standard Matlab function normfit was used to compute the standard deviations of ${{\theta }}_{ij}$ and ${\hat{\theta }}_{ij}$ with their 95% confidence intervals, which were taken as the measure of the certainty of the localisation with and without individual bias, respectively. The same Matlab function was also used to compute the mean value from absolute error measures ${{\phi }}_{ij}$ and ${\hat{\phi }}_{ij}$. These values were taken as measures of the directional accuracy of perception obtained in general with the system.

It could be argued, that the subjects may have memorised the individual sound spectra produced by the sources, as the piezo drivers produced somewhat different spectra, and the subjects were allowed to listen to each source separately as part of the familiarisation step. However, the non-compensated distribution of angular errors in Fig. 2 shows that the frequency of selecting the actual source was 21.7% and the frequency of selecting the next-left or next-right sources were 20.4% and 19.4%, respectively, whereas the success rate based upon guesswork alone, would be 12.5%. Furthermore, if the identification would have been based on comparison to memorised source spectra, the sources immediately adjacent to the actual source would have no preference in the test over other potential sources. It can therefore be counter argued, that the perceived direction was indeed the predominant information processed by the subjects to identify which source was emitting the ultrasonic sound.

## Supplementary information

Supplementary Video 1.

Supplementary Video 2.

Supplementary Video 3.

Supplementary Video 4.
